# Characterization of Vitronectin Effect in 3D Ewing Sarcoma Models: A Digital Microscopic Analysis of Two Cell Lines

**DOI:** 10.3390/cancers16193347

**Published:** 2024-09-30

**Authors:** Amparo López-Carrasco, Karina Parra-Haro, Isaac Vieco-Martí, Sofía Granados-Aparici, Juan Díaz-Martín, Carmen Salguero-Aranda, Delia Acevedo-León, Enrique de Álava, Samuel Navarro, Rosa Noguera

**Affiliations:** 1 Incliva Biomedical Health Research Institute, 46010 Valencia, Spain; malopez@incliva.es (A.L.-C.); iviemar@alumni.uv.es (I.V.-M.); sgranados@incliva.es (S.G.-A.); samuel.navarro@uv.es (S.N.); 2 Centro de Investigación Biomédica en Red de Cáncer, Instituto de Salud Carlos III, 28029 Madrid, Spain; jdiaz-ibis@us.es (J.D.-M.); csalguero-ibis@us.es (C.S.-A.); enrique.alava.sspa@juntadeandalucia.es (E.d.Á.); 3 Pathology Department, Medical School, University of Valencia, 46010 Valencia, Spain; kapaha@alumni.uv.es; 4 Instituto de Biomedicina de Sevilla, Department of Pathology, Hospital Universitario Virgen del Rocío, CSIC-Universidad de Sevilla, 41013 Seville, Spain; 5 University Hospital Dr. Peset, 46017 Valencia, Spain; acevedo_del@gva.es; 6 Department of Normal and Pathological Cytology and Histology, School of Medicine, University of Seville, 41009 Seville, Spain

**Keywords:** extracellular matrix, hydrogels, digital quantification, childhood cancer

## Abstract

**Simple Summary:**

Three-dimensional (3D) models enable simplified and controlled in vitro replication of tumor microenvironment features, making them easier to study. In the current work, we developed 3D hydrogels based on gelatin plus silk fibroin with tunable mechanical properties, incorporating vitronectin (a glycoprotein related to tumor aggressiveness) into the scaffolds. Digital analysis was applied to monitor Ewing sarcoma cell line growth and morphology in response to the 3D model composition and different culture times, opening the possibility of its use to study other proteins of interest and tumor types. Our findings highlight the heterogeneity of cell line responses to their microenvironment, underscoring the enormous potential of these hydrogels as mechanodrug-testing platforms and for studying cell protein secretion as an initial step in liquid biopsy biomarker research.

**Abstract:**

Ewing sarcoma (ES) is an aggressive bone and soft-tissue pediatric cancer. High vitronectin (VN) expression has been associated with poor prognosis in other cancers, and we aimed to determine the utility of this extracellular matrix glycoprotein as a biomarker of aggressiveness in ES. Silk fibroin plus gelatin–tyramine hydrogels (HGs) were fabricated with and without cross-linked VN and cultivated with A673 and PDX73 ES cell lines for two and three weeks. VN secretion to culture media was assessed using ELISA. Morphometric analysis was applied for phenotypic characterization. VN release to culture media was higher in 3D models than in monolayer cultures, and intracellular, intercellular, and pericluster presence was also observed. A673-HGs showed lower density of clusters but a proportion of larger clusters than PDX73-HGs, which presented low cluster circularity. The cluster density of A673-HGs without added VN was higher than with added VN and slightly lower in the case of PDX73-HGs. Furthermore, a culture time of three weeks provided no benefits in cluster growth compared to two weeks, especially in A673-HGs. These advances in 3D modeling and digital quantification pave the way for future studies in ES and other cancers to deepen understanding about intra- and intercellular heterogeneity and anti-adhesion VN therapies.

## 1. Introduction

Ewing sarcoma (ES) is the second most frequent bone or soft-tissue cancer in children, adolescents, and young adults [1,2]. The exact origin of ES is still under debate, but mesenchymal stem cells (MSC) are currently considered the most likely candidates [3,4,5]. Histologically, ES is characterized by sheets of small, round, and uniformly blue tumor cells with round nuclei and limited cytoplasm. These cells display morphological characteristics indicative of neural differentiation, such as rosette formation [5,6]. Standard diagnostic practice consists of immunohistochemical staining for the membrane markers CD99 and NKX2.2 and evaluation for loss of neural differentiation [2].

ES is well characterized at the molecular level and is mainly defined by recurring translocations that generate oncogenic gene fusions involving the *FET* gene family (*EWSR1*, *FUS*, *TAF15*) as 5′ gene fusion partners, with *EWSR1* being the common partner in most cases. It also involves members of the *ETS* gene family of transcription factors as 3′ partners, particularly *FLI1* (in about 85% of cases), *ERG* (around 10%), and occasionally other variants such as *ETV1*, *ETV4*, or *FEV* (less than 1% of cases) [2]. Concurrent chromosomal copy number alterations are also observed, including gains on chromosomes 1q, 8, 12, or 20 and losses on 16q [2,3,7]. 

The current standard treatment for ES is a multimodal approach consisting of conventional chemotherapy and surgery established through collaborative international trials, which has led to an improved overall survival (OS) rate of approximately 85% [6,8]. In contrast, patients with metastatic disease at diagnosis present survival rates of less than 30% [2,6]. A fuller understanding of the prognostic factors that influence treatment outcomes poses a significant clinical challenge, and this raises a need for more in-depth exploration of novel diagnostic and therapeutic targets [5]. 

The tumor microenvironment (TME) plays a critical role in the effectiveness of cancer treatments, particularly immunotherapy. It can influence tumor behavior and aggressiveness due to the remarkable adaptability of tumor cells and intratumor heterogeneity [9]. Within the TME, the extracellular matrix (ECM) forms a complex network of fibrillar proteins, glycoproteins, and proteoglycans, which act as the scaffold for cellular embedding [10,11]. Cells connect their cytoskeleton to the ECM through receptors such as integrins, thereby generating biophysical forces (traction and compression) known as biotensegrity [12]. This leads to the transmission of biochemical signals via mechanotransduction processes and ultimately affects gene expression and cell survival, progression, and migration [13,14,15]. Moreover, mechanotransduction also induces feedback mechanisms that reciprocally influence ECM composition and architecture. This results in clonal selection of the tumor cells best adapted to the niche [16,17,18]. 

In the context of ES, it is plausible that changes in the mechanical properties of the ECM contribute to the abnormal growth and ability to spread of these cells. Previous studies with ES have shown increased synthesis of ECM proteins in response to activation of the Wnt pathway [19], and TAZ/YAP1 proteins, which are involved in sensing mechanical signals, have also been observed to play a role in ES progression [20,21]. These findings have raised the possibility of developing new therapeutic strategies targeting different players in the TME of bone ES [22].

Among the components of the ECM, vitronectin (VN) is a glycoprotein also present in plasma, which is secreted as a monomer or multimer. It contains multiple binding sites to cells, including integrins and other membrane receptors, such as uPAR and PAI-1 [23], and to ECM components (fibers and proteoglycans), creating temporary connections and facilitating cell adhesion and migration [23,24,25]. VN has been implicated in tumor progression and metastasis in various types of cancer, including osteosarcoma [26], melanoma [27], hepatocarcinoma [28], breast cancer [29], lung cancer [30], and ovarian cancer [31]. In the childhood cancer neuroblastoma (NB), the presence of VN in biopsy samples has been associated with poor prognosis [32]. VN expression and its relation to aggressiveness have been poorly studied in ES. In a preliminary investigation, our group described VN expression in 16 Formalin-Fixed Paraffin-Embedded (FFPE) samples derived from original ES tumors [33], observing in 7 of these samples a branching in VN expression reminiscent of track formation, which could facilitate migration and metastasis.

Given the rarity of childhood tumors, and in line with the ethical commitment to reducing animal experimentation, other material source options need to be explored for ES research. Three-dimensional (3D) tumor models have become valuable tools offering promising advantages for cancer research [16,34,35]. These biomaterial-based models surpass traditional 2D cultures by reproducing several features of the complex ECM, allowing controlled studies of biotensegrity and intratumor heterogeneity [36]. Moreover, 3D models have served as testing platforms for new drugs [34,35,37]. Despite this, the use of these 3D models, including free and hydrogel (HG)-based spheroids, scaffold constructs, and flow perfusion systems [38,39], is not yet widespread in ES research. 

In previous studies, we used innovative 3D models of NB such as bio-printed HGs with methacrylated gelatin and methacrylated alginate with varying degrees of stiffness and polyethylene glycol HGs with cross-linked VN [10,12,16,22,40]. More recently, we used silk fibroin with tyramine–gelatin (sf-GTA)-based HGs, also with and without cross-linked VN with four different NB cell lines (the commercial SK-N-BE(2) and SH-SY5Y cell lines and two patient-derived xenograft [PDX] cell lines) [41].

Our aim in this study was to determine whether two different ES cell lines would be able to adapt and grow in sf-GTA 3D models. We also explored the effect of cross-linking VN in 3D models on cell growth, morphology and VN expression and secretion to the ECM of the ES cell lines and compared these results with those in previous 2D cultures. Finally, we assessed potential applications of these HG models for future research.

## 2. Materials and Methods

### 2.1. Monolayer Cultures (2D)

The cell lines used were commercial ES cell line A673 (derived from the muscle cancer cells of a 15-year-old girl in 1973, ATCC, Masassas, VA, USA) and ES cell line PDX73 (taken from the metastatic costal tumor of a 16-year-old girl in 2015, Hospital del Rocio, Sevilla). Both cell lines possess the diagnostic genetic fusion EWSR1-FLI1. The gain of chromosome 8 and loss of the 16q region associated with poor prognosis is present in the PDX73 but not the A673 cell line [42]. Gibco™ DMEM medium (Gibco, Life Technologies, Waltham, MA, USA) and Ham’s F-10 Nutrient Mix, GlutaMAX medium (Gibco, Life Technologies, Waltham, MA, USA) were used for A673 and PDX73 cultures, respectively, supplemented with 10% fetal bovine serum and 1% penicillin/streptomycin. Cell cultures were maintained at 37 °C with 5% CO_2_ in T75 cell culture flasks (plus 0.1% gelatin coated in the case of PDX73 to facilitate cell adhesion). The media were changed every 2–3 days, and cultures were trypsinized when confluence was reached.

### 2.2. Hydrogel Cultures (3D)

We manufactured the 3D HGs of GTA and sf following a previously described protocol [43] in which the methodology of the model was established and the authors cultured one human bone marrow mesenchymal stem cell line (hMSCs). A solution of lyophilized GTA (Sigma Aldrich, Merck, St. Louis, MO, USA) and sf (Sigma Aldrich, Merck, St. Louis, MO, USA) at a ratio of 25:75, respectively, was dissolved in IMDM (4% *w*/*v*). VN (PrepoTech, Rocky Hill, NJ, USA) was resuspended in dPBS and combined with the sf-GTA mix with a final concentration of 0.4 mg/mL for the HGs with added VN; the same amount of dPBS alone was added for the HGs without added VN. Horseradish peroxidase (20 U/mL) was also added to the mix. Cells were trypsinized from the previous 2D culture, quantified with trypan blue using a TC20 Automated Cell Counter (Bio Rad, Hercules, CA, USA), and resuspended in the HG mix to obtain an amount of 1.25 × 10^5^ commercial cells or 2.5 × 10^5^ PDX cells per HG. To start the polymerization process, 2 µL of hydrogen peroxide (0.01%) was placed in the center of each well in a 24-well plate and mixed with aliquots of 60 μL of the mix solution. HGs were maintained in the incubator for 1 h until complete polymerization, then 2 mL of supplemented culture medium (same as that for the 2D cultures) was added over each HG. The HG culture medium was replaced every 3 days during the 2 and 3 weeks that the 3D models were maintained. Three HG replicates of each composition and culture time were cultivated with each cell line. The waste medium was frozen (−80 °C) and preserved for ELISA. 

### 2.3. Two-Dimensional and Three-Dimensional Sample Preparation for Digital Analysis

Once the monolayer culture of each line reached cell confluence, a 250 μL aliquot containing approximately 100,000 cells was taken to perform the cytospin procedure. Slides were kept in ethanol until staining. HGs were FFPE after 2 and 3 weeks of culture, then cut into 3 µm sections. Both cytospin and HGs section samples were stained with H&E and immunostained with anti-VN rabbit monoclonal antibody (EP873Y, Clone; ab45139, Abcam, Cambridge, MA, USA) diluted at 1:100, using OptiView Amplification Kit (Ventana Medical Systems Inc., Tucson, AE, USA) in the BenchMark XT automated slide staining system (Ventana Medical Systems Inc., Tucson, AE, USA). The mounted plates were scanned with Ventana iScanHT (Roche, Basel, Switzerland) at 20× and the resulting images were saved in TIF format. Cell and cluster quantity and morphology in H&E and anti-VN stainings from 2D and 3D models were subjectively evaluated by expert observers, including the reference pathologist of the group (SN), who also validated the objective digital analyses. As previously mentioned, the nomenclature for VN expression was adopted from Burgos-Panadero’s research on NB [32]. It was classified into two distinct categories: strong VN intensity inside cells or immediately adjacent to the cell membrane was termed territorial VN and weak to moderate VN intensity in intercellular location was interterritorial VN. A third category, named pericluster VN, was established to designate the strong VN intensity detected at the edge of the clusters, just between the cells and the scaffold, as well as in the scaffold itself close to the clusters forming as a crown. Some clusters of HGs with added VN showed reduced VN intensity in the scaffold around them (a weak stain) that we refer to as a pale halo. 

### 2.4. Digital Microscopic Analyses

QuPhat^TH^ (Belfast, Northern Ireland, UK) and its extensions was used for all digital analyses [44]. A StarDist script was adjusted to detect VN positivity in cytospin of 2D cultures, based on the diaminobenzidine mean intensity threshold in cytoplasm. For digital analyses of 3D models, a semi-automatic method was developed to detect HG area, clusters, and cell counts in the scanned H&E-stained slides (Supplementary Materials Section S1, Figure S1). In total, 11 H&E stain images were analyzed for A673 (one image was excluded from the study because the HG was broken into multiple fragments during FFPE processing and the holes and folds precluded correct analysis) and 12 for PDX73. To evaluate cluster density, the number of clusters divided by HG area of each replicate was analyzed. We used quartile analysis of the cell count in each cluster of the ES cell lines, and a consensus was reached for cluster size classification (Table 1). Cluster area and circularity were parameters provided by QuPath^TH^. Clusters with a more circular shape have a circularity value closer to 1, meaning that fewer cell protrusions are present. Therefore, an irregular shape will have reduced circularity.

### 2.5. Detection of VN in Culture Media

For 2D cultures, medium (8 mL) was collected from the confluent culture in a T75 cell culture flask after quantifying the culture cells with TC20 Automated Cell Counter. The medium from 2D cultures was concentrated 16-fold with SpeedVac™ (Thermo Fisher Scientific, Newington, NH, USA). In the 3D cultures, culture media from two HG replicates (4 mL) of each composition and time of culture were mixed and concentrated 8-fold. With this step, we reduced the variability intrinsic to HG replicates and achieved the minimum VN level for ELISA detection (assay sensitivity of 15.19 pg/mL). As culture media were collected with each change (every 3 days), we analyzed VN levels with ELISA at several time points. However, the cell count was estimated in both HGs at 2-week and 3-week time points (when HGs were FFPE) by digital analysis in 3 µm sections, then was scaled to the approximate volume of HGs (28 mm^3^) and added. VN level detected by ELISA in the 2D and 3D culture media was divided by concentration factor and by cell count to determine VN secretion in ng/mL per million cells. The amount of VN in culture media was determined by sandwich colorimetric enzyme-linked immunosorbent assay (ELISA, Novus Biologicals, Centennial, CO, USA) according to the manufacturer’s instructions. Antibody reactions, absorbance reading and results calculation steps were performed using a Triturus automated analyzer (Grífols, Barcelona, Spain). 

### 2.6. Statistical Analysis

The database used was obtained from QuPath^TH^. Statistical analyses were performed with R studio version 4.2.1 (Foundation for Statistical Computing, Vienna, Austria), using the ggplot2 package version 3.5.1 (Wickham, 2016) to generate figures, and GraphPad Prism 8 (Graphpad Software, Boston, MA, USA). Data were analyzed for normality by Shapiro–Wilk test, and then the Kruskal–Wallis method with Dunn’s test was used for nonparametric comparisons of cluster density, area, and cluster circularity between cell lines, culture times, and HG compositions. Differences were considered if the *p*-value was <0.05.

## 3. Results

### 3.1. VN Cell Expression and Secretion to Culture Media in 2D Models

**Figure 1:**
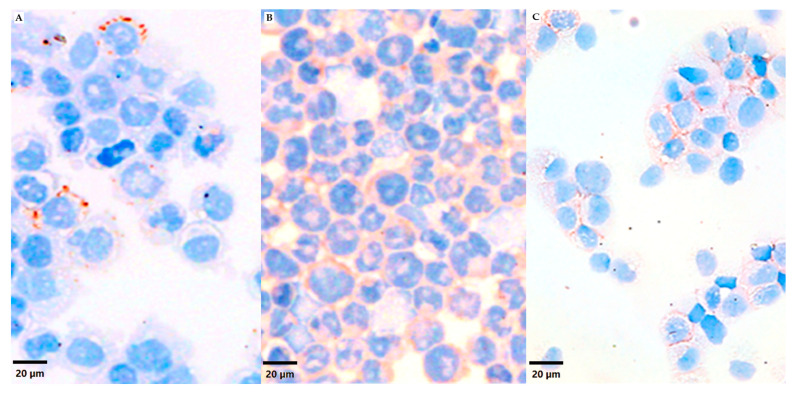
Vitronectin (VN) expression of Ewing sarcoma (ES) and neuroblastoma (NB) cell lines in 2D cell cultures. Cytospins from 2D cultures of A673 (**A**) and PDX73 (**B**) and SH-SY5Y (**C**) cell lines, immunostained with anti-vitronectin antibody, are shown in 20 µm viewer scale.

Anti-VN staining revealed different VN expression patterns between the A673 and PDX73 cell lines cultured in monolayer in the cytospin area (approx. 2 cm^2^). We quantified 71,776 cells in the cytospin of the A673 cell line, finding a 12.31 µm cell diameter and 0.68 nuclei/cell ratio. VN expression was low (5.13% of positive cells) and was characterized by a granular staining pattern in some cells (Figure 1A). In contrast, we quantified 131,821 cells with a 13.01 µm cell diameter and 0.73 nuclei/cell ratio in the cytospin of the PDX73 cell line. These cytospins showed positivity in many cells (76.67%) with weak intensity in the cytoplasmatic submembrane compartment (Figure 1B). The difference in the positive cell percentage and the different morphology of VN cell storage in ES was also found in NB cells. An image of NB (cell line SH-SY5Y) included as a control sample of VN expression heterogeneity showing 50% positivity only in the cellular membrane can be found in Figure 1C.

VN release to culture media of the two Ewing sarcoma cell lines was detected by applying ELISA (Figure S2). In correlation with our immunohistochemical cell expression findings, the A673 cell line cultured in monolayer showed a lower VN release to culture media per million cells (1.2 ng/mL) compared to the PDX73 cell line (1.9 ng/mL) and SH-SY5Y cell line (1.8 ng/mL), although the cell count in 2D cultures was higher for A673 than for the PDX73 or SH-SY5Y cell lines. 

### 3.2. VN Cell Expression and Secretion to the ECM in 3D Models

**Figure 2:**
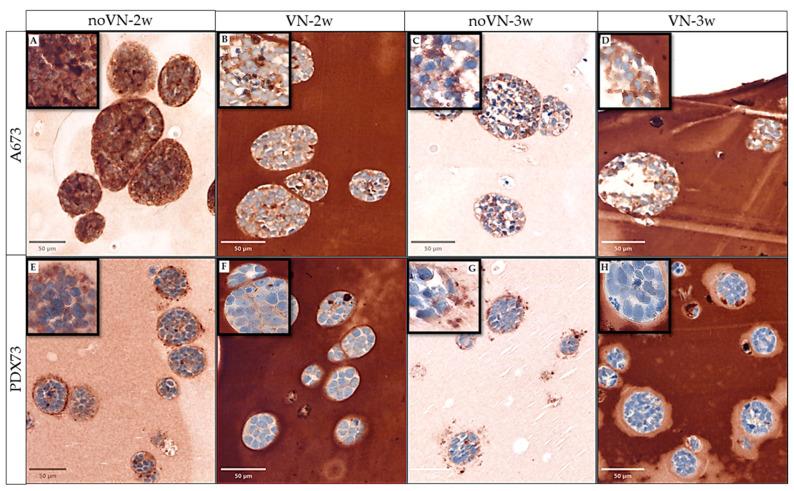
Vitronectin (VN) expression of Ewing sarcoma (ES) cell lines in 3D cell cultures. Digital images of VN expression of ES cell lines A673 (**A**–**D**) and PDX73 (**E**–**H**) grown in hydrogels (HGs) without and with added VN (50 µm viewer scale) and zoom zones in a 10 µm viewer scale (top left squares). (**A**,**D**) Non-added-VN HGs at 2 weeks (2w). (**B**,**E**) Added-VN HGs at 2w. (**C**,**F**) Non-added-VN HGs at 3 weeks (3w). (**G**) Added-VN HGs at 3w culture.

As already noted with 2D cultures, in 3D cultures, different VN expression patterns were observed between the two ES cell lines (Figure 2). Subjective evaluation of VN immunostainings revealed that in A673 HGs without added VN at 2 weeks of culture, cells exhibited the highest expression intensity (+++), uniformly distributed, and that VN was primarily stored intracellularly and to a lesser extent in the pericellular region [territorial VN] (Figure 2A). At 3 weeks with the same scaffold composition (HGs without added VN) and at both culture times in HGs with added VN (Figure 2B–D), similar intensity and location of VN expression was detected; the intensity was moderate (++) and more prevalent in the intercellular zone (interterritorial VN) and adjacent to the edge between the cluster cells and the scaffold. 

PDX73 cells at 2 and 3 weeks without added VN (Figure 2E,G) exhibited moderate (++) interterritorial staining intensity and particularly pericluster expression, and we were surprised to observe a marked VN pattern close to the cluster contour (as a spiked crown), especially at 2 weeks. In HGs with added VN, there was low-intensity interterritorial VN (+) without a clearly discernible crown at 2 weeks (Figure 2F). Curiously, at 3 weeks (Figure 2H), in HGs of the same composition (with added VN) there was almost no intra- or interterritorial VN expression; however, a pale halo was visible around the clusters, lighter than the rest of the HG stain, with an irregular shape and sometimes much thicker on one side of the cluster than the other.

VN release to 3D culture media was null in control HGs (without cells) with added VN after both 2 and 3 weeks of culture. However, culture media of both ES cell lines grown in 3D showed positive VN detection. Overall, VN was higher in 3D than in 2D cultures of both cell lines (*p* = 0.044, Figure S3).

Similar VN release was found at 2 and 3 weeks without added VN in both cell lines (Figure S4). VN cross-linking in HGs did not prevent VN secretion by ES cells, which remained elevated despite the large quantities present in the scaffold. Longer culture times (3 weeks) in HGs with VN cross-linking decreased cell growth in the A673 cell line but increased VN release, coinciding with the lower amount of intracellularly retained VN observed in immunostaining. Longer culture time slightly increased PDX73 cell numbers, and VN secretion to culture media remained elevated under all the 3D conditions studied.

### 3.3. Digital Analisis and Morphometric Parameters of Clusters and Cells of 3D Models

Cluster density, cell density by cluster size, cluster area, and circularity of clusters were measured by digital analysis and validated subjectively by experts. 

**Figure 3:**
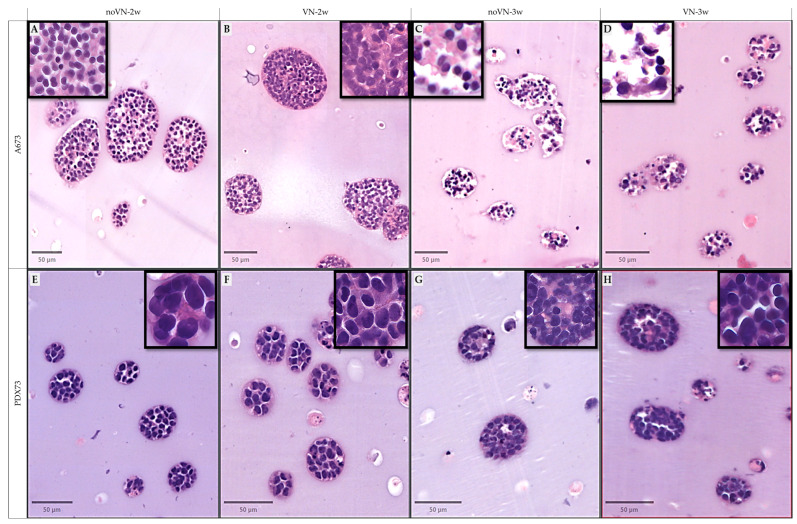
Digital imagen of examples of clusters in hydrogels (HGs) stained with hematoxylin and eosin. (**A**–**D**) HGs of cell line A673, (**E**–**H**) HGs of cell line PDX73, (**A**,**E**) non-added VN at 2 weeks (2w), (**B**,**F**) added VN at 2w, (**C**,**G**) non-added VN at 3w, (**D**,**H**) added VN at 3w of culture. In 50 µm viewer scale and zoom zones in 10 µm viewer scale (Top squares).

Subjective observation revealed that at 2 weeks of culture, in approximately 95% of clusters of the A673 cell line grown in HGs without and with added VN, the cells displayed large nuclei with varying hematoxylin intensity and evident cytoplasm (medium to high eosinophilic), with reduced intercellular spaces (Figure 3A,B). In contrast, after 3 weeks of culture, in both scaffolds (HGs without and with added VN; Figure 3C,D) approximately 80% of clusters contained fewer cells, each with small nuclei, reduced cytoplasm, and wide intercellular spaces. All PDX73 clusters showed similar cell morphology regardless of culture time or scaffold composition (2/3 weeks of culture and without/with added VN). These cells had bigger nuclei than in the A673 line, with great variability in hematoxylin intensity, creating the so-called dark and light cells. The cytoplasm was generally thin, with limited intercellular spaces, and minimal eosinophilic material (Figure 3E–H).

We used data from a total of 8665 clusters and 175,227 cells from the 23 H&E images (Table S1) for digital analysis. Note that there was variability in the number and size of clusters between HGs replicates (Figure S5). The median area of the HGs sections was 8.2 cm^2^ (between 3.99 cm^2^ and 20.85 cm^2^). Irrespective of scaffold composition and time of culture, the A673 cell line exhibited significant heterogeneity in the cellular features in clusters and a larger cluster size compared to the PDX73 cell line, which showed uniform cellular characteristics with smaller cluster sizes. Specifically, in the A673 cultures, we detected 727 clusters in the three HGs replicates without added VN at 2 weeks (median cluster area = 1358.7 µm^2^), 524 clusters in the three HGs with added VN at 2 weeks (median cluster area = 1385.7 µm^2^), 269 clusters in HGs without added VN at 3 weeks (median cluster area = 719.21 µm^2^), and 483 clusters in HGs with added VN at 3 weeks (median cluster area = 925.085 µm^2^). In the case of the PDX73 cell line, 1709 clusters were found in HGs without added VN at 2 weeks (median cluster area = 595.43 µm^2^), 1708 clusters in HGs with added VN at 2 weeks (median cluster area = 437.12 µm^2^), 1505 clusters in HGs with added VN at 3 weeks (median cluster area = 457.49 µm^2^), and 1740 clusters in HGs without added VN at 3 weeks (median cluster area = 488.44 µm^2^). 

#### 3.3.1. Cluster Density 

**Figure 4:**
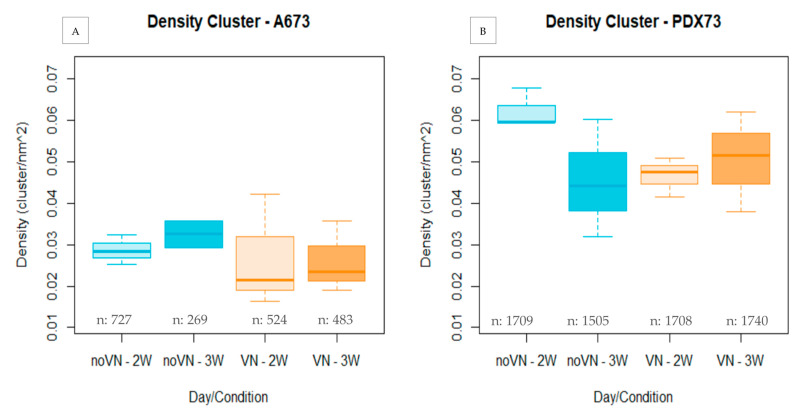
Cluster density (clusters/nm^2^) in 3D cultures evaluated by hydrogel composition and time of culture. (**A**) A673 and (**B**) PDX73 cell cultures. Boxes in cyan represent non-added VN in the scaffold (noVN), boxes in orange represent added VN in the scaffold (VN), light colors represent 2-week culture (2w) and dark colors represent 3-week culture (3w), n: refers to the number of clusters detected in the hydrogels of each composition and time of culture.

The A673 showed lower cluster density (median = 0.028 cluster/nm^2^) than the PDX73 cell line (median = 0.051 cluster/nm^2^, *p* < 0.0001, Figure S6) in both scaffolds and culture times. Specifically, cluster density of the A673 cell line (Figure 4A) was higher when cultured in HGs without added VN than with added VN (median = 0.0302 cluster/nm^2^ vs median = 0.0263 cluster/nm^2^, respectively) at both short and long culture times. Moreover, we detected a slight increase in cluster density with time of culture (median = 0.028 clusters/nm^2^ at 2 weeks vs. median = 0.0325 clusters/nm^2^ at 3 weeks in HGs without added VN, and median = 0.0215 clusters/nm^2^ at 2 weeks vs. median = 0.0234 clusters/nm^2^ at 3 weeks in HGs with added VN).

Cluster density of the PDX73 cell line (Figure 4B) was highest in scaffolds without added VN at short culture time but at long culture time was highest in scaffolds with added VN. The PDX73 cell line showed notably higher cluster density in HGs without added VN than in HGs with VN cross-linking at 2 weeks (median = 0.060 cluster/nm^2^ vs. median = 0.046 cluster/nm^2^, respectively). At the longest culture time, however, the reverse situation was observed. Cluster density was lower in HGs without added VN than in the HGs with added VN, reflecting a better adaptation to these scaffolds over time (median = 0.048 cluster/nm^2^ vs. median = 0.051 cluster/nm, respectively). 

##### Cluster Density Classified by Cluster Size

Cluster density was also calculated after classifying clusters by cell count following the established criteria (Table 1, Section 2). 

**Figure 5:**
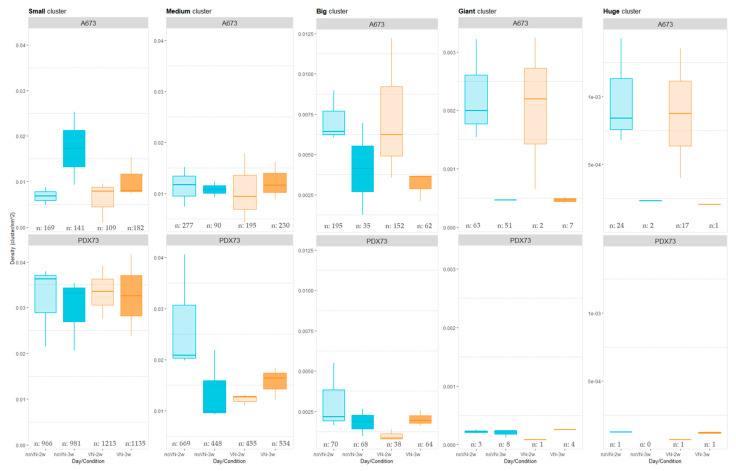
Cluster density (clusters/nm^2^) in 3D cultures classified by cluster size. The top graphs indicate the A673 cell line and bottom graphs the PDX73 cell line. Boxes in cyan represent non-added VN in the scaffold (noVN), boxes in orange represent added VN in the scaffold (VN), light color shading represents 2-week cultures (2w) and dark colors represent 3-week cultures (3w), n: refers to the number of clusters of each size detected in the hydrogels of each composition and time of culture. Note the changes in density scale between cluster sizes, as indicated in the Y-axis of the graphs.

A673 showed higher density in the largest clusters (big, giant, and huge) than PDX73, which exhibited more density for the smallest (small and medium) ones (Figure 5). Specifically, cell line A673 (Figure 5, top graphs) showed no remarkable differences in any size category with respect to VN cross-linking in the scaffolds. Regarding time, we observed higher cluster density in the largest clusters at 2 than at 3 weeks of culture. However, small clusters increased their density with time. Classifying the clusters of PDX73 HGs by size (Figure 5, bottom graphs), we found only subtle differences between the two scaffold compositions and the two culture times studied, with more variability present in the medium cluster density. 

#### 3.3.2. Cluster Areas 

**Figure 6:**
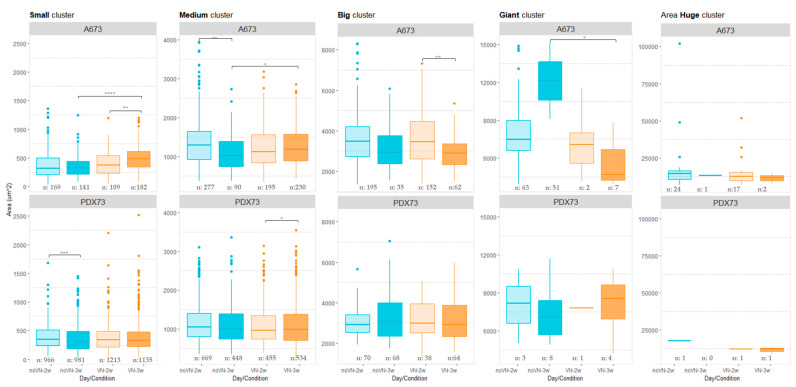
Cluster area (μm^2^) in 3D cultures classified by cluster size. Top graphs refer to A673 cell line and bottom graphs to PDX73 cell line. Boxes in cyan represent non-added VN in the scaffold (noVN), boxes in orange represent added VN in the scaffold (VN), light colors represent 2 weeks cultures (2w) and dark colors represent 3 weeks cultures (3w), n: refers to the number of clusters of each size detected in the hydrogels of each composition and time of culture. Kruskal–Wallis method with Dunn´s test revealed significant differences: * = *p* < 0.05, ** = *p* < 0.01, *** = *p* < 0.001, **** = *p* < 0.0001. Note the changes in area scale between cluster sizes, as indicated in the Y-axis of the graphs.

The high variability in A673 cell line cluster areas (Figure 6, top graphs), despite previous classification by cell count, is partly due to cell size and intercellular spaces (Figure 3). Briefly, the small and medium clusters of A673 in HGs without added VN at 3 weeks showed a significantly lower area than the scaffolds with added VN (*p* < 0.0001 for small and *p* = 0.017 for medium clusters), the latter ones also showing an increase in small cluster areas with time (*p* = 0.0010). Among the big clusters, the HGs with added VN showed a significant area decrease with time (*p* = 0.0051). Finally, the giant clusters, as opposed to the rest of the cluster sizes, had a bigger area in HGs without than with added VN at 3 weeks (*p* = 0.021). 

Validated subjectively, the PDX73 cell line (Figure 6, bottom graphs) showed more stable cluster areas between conditions and slightly smaller cluster area sizes than the A673 cell line (Figure 3). However, the small clusters showed a decrease in area in the non-VN-added scaffolds over time (*p* = 0.0008), while in the medium clusters, the area was significantly lower at 2 than at 3 weeks within VN-added HGs (*p* = 0.0124). Data regarding the cluster area of the two ES cell lines are summarized in Tables S2 and S3.

#### 3.3.3. Circularity of Clusters

**Figure 7:**
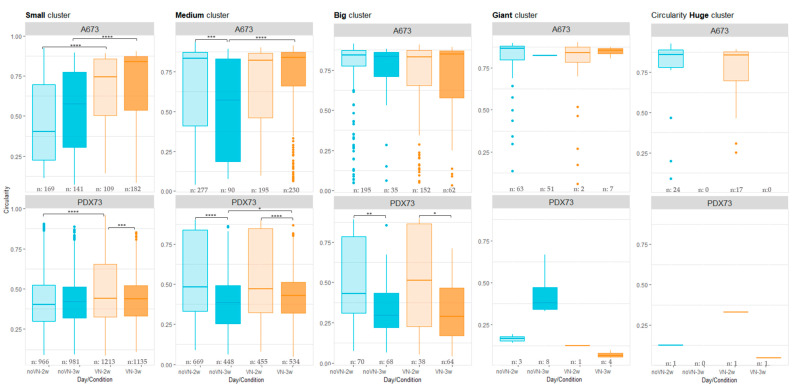
Cluster circularity in 3D cultures classified by cluster size. The top graphs show the A673 cell line and bottom graphs the PDX73 cell line. Boxes in cyan represent no added VN in the scaffold (noVN), boxes in orange represent added VN in the scaffold (VN), the lighter shading of each color representing culture for 2 weeks (2w) and the darker shading culture for 3 weeks (3w), n: refers to the number of clusters of each size detected in the hydrogels of each composition and time of culture. Kruskal–Wallis method with Dunn´s test revealed significant differences: * = *p* < 0.05, ** = *p* < 0.01, *** = *p* < 0.001, **** = *p* < 0.0001. Clusters with a more circular shape have a circularity value closer to 1.

The A673 cell line (Figure 7, top graphs) showed higher circularity than PDX73 for all cluster size categories. The circularity of the small clusters was lower in HGs without added VN than in VN-added scaffolds at both timepoints, 2 weeks (*p* < 0.001) and 3 weeks (*p* < 0.001). The medium clusters showed an irregular shape at 3 weeks compared to 2 weeks in non-added VN HGs (*p*-value = 0.009) and when compared with the VN-added HGs 3 weeks (*p*-value < 0.001). The largest clusters showed circularities closer to 1 in all the A673 culture conditions studied (Tables S4 and S5).

High irregularity was detected, mainly in all cluster sizes in the 3D models of PDX73 (Figure 7, bottom graphs). Small, medium, and large clusters showed a similar pattern. They presented a similar or lower irregularity at 3 weeks of culture than at 2 weeks, which varied depending on the composition of the HGs (Figure 7). The largest clusters showed more reduced circularity than the smaller ones. Particularly notable was a higher circularity of giant clusters in non-VN-added scaffolds at 3 weeks compared to the other conditions. Tables S6 and S7 show comparison of circularity of PDX73 clusters between cluster size categories in each 3D culture condition. 

## 4. Discussion

ES is considered a cancer type with poor prognosis. Its clinical characteristics often result in late diagnosis after spread to metastatic sites, thus limiting effective treatment [45]. Chemotherapy resistance is closely linked to specific TME–cell interactions in ES [46], and the influence of biotensegrity and mechanical alterations of the ECM facilitates cancer cell proliferation and dissemination [9,47]. Previous studies have shown that primary tumor stiffness is greater than in normal tissues and has been linked to cancer progression and metastasis [32,48,49]. This has prompted researchers to develop alternatives for new therapies, including mechanotherapies, aimed at modulating mechanical forces, for example, by disrupting communication between tumor cells and ECM components and thus inhibiting migration [50]. Simplified replication of the TME through 3D models is essential to improve our understanding of tumor aggressiveness, progression, and migration and can potentially be used to develop new diagnostic biomarkers and test therapeutic strategies. However, 3D models are as yet underutilized in ES research; the complexity, cost, and technical challenges of developing these systems represent obstacles to scalability and broader adoption [38,39]. 

Our research is focused on alternative 3D models that are simple and quick to manufacture in terms of composition and structure. We designed HG models comprising sf-GTA, with or without VN cross-linking. This glycoprotein was chosen for its overexpression and implication in several cancer types associated with tumor aggressiveness [26,28,29]. The constructed HGs allowed us to simulate 3D tumors with two mechanical forces, induced by the scaffolds and by the cell dynamic related to the VN amount added and/or secreted by cells. Since VN cross-linking increases matrix stiffness, promoting migration and proliferation [12,51], we hypothesized that cross-linking VN to the HGs would activate more mechanotransduction pathways in cells through ECM–cell interaction [2,52]. 

Cell growth and morphology in the 2D and 3D cultures were monitored and evaluated using digital pathology analyses. The integration of artificial intelligence (AI) applied to digital pathology has proven crucial in oncology research [53], providing new approaches for preclinical studies which enable us to evaluate not only cell features but also the composition and architecture of the ECM [52], through which new biomarkers can be assessed. This integration optimizes analysis and reduces the time pathologists need to perform more precise evaluations [54], thereby enhancing diagnostic, prognostic, and predictive clinical decision-making in ES and other tumors [40].

When we scaled from the 2D cultures, we observed that the biocompatibility and tensegrity of the constructed ECM provided a suitable environment for the two ES cell lines, A673 and PDX73, to grow and lead cluster formation and VN secretion.

Positive detection of VN secreted to 2D and 3D culture media indicated that both cell lines synthesized this glycoprotein in all assayed conditions, even in the presence of cross-linked VN in the HG [55,56]. Nonetheless, VN release was higher in 3D than 2D cultures, reflecting the importance of cell growth in a more biomimetic 3D environment. 

The lower amount of VN secreted by A673 than PDX73 cells in 2D culture correlates with less adaptation to 3D culture. As the A673 cell line (established in 1973) has undergone numerous 2D culture passages, the cells have experienced several transformations, new mutations, and loss of their original flexibility. The low percentage of cells with VN granules must have quickly and intensely released their concentrated content to the culture medium, given the unexpected difference with the other cell lines analyzed. When A673 cells were cultured in the 3D models, VN staining intensity became higher and more uniform in a high number of clusters, which could be related to the clonality of the secreted cells. The PDX73 cell line, a short-term culture (established and frozen in 2015) with no more than 13 passages in 2D culture, may have better retained the characteristics of the original tumor with a less variability in VN release and cellular expression, and more homogeneous and diffuse staining in 2D and 3D cultures. In this cell line, we validated previously described evidence that VN is more uniformly distributed on the cell membrane due to a more complex environment in which focal adhesions are continuously formed [17,34]. How this VN secretion by 3D-cultured ES cells may be reflected in the blood plasma of patients with these tumors is an interesting future line of study. Elevated VN levels have previously been described in plasma (and other fluids) of poor prognosis patients with glioma [57], melanoma [27], and breast [58], ovarian, and endometrial cancer [59] and in children with Hodgkin’s lymphoma [60], acute lymphoblastic leukemia [61], and NB [41]. The use of circulating biomarkers in liquid biopsy is becoming increasingly widespread due to its simplicity and speed, and they play an especially important role in cancers such as ES to avoid invasive biopsies in children and because of the high intratumoral heterogeneity of these tumors, as is clearly reflected in the 3D models presented here.

Although both cell lines exhibited the phenotypic trait of small, round, and blue cells of bone and soft tissue sarcomas [2], 2D and 3D culture analysis with anti-VN and H&E stains revealed differences in their morphology and biological behavior. Taking all data together, the A673 cell line showed lower cluster density but larger cluster sizes than PDX73 cells. However, our results revealed that VN presence in scaffolds had a differential effect on cluster proliferation in both A673 and PDX73 cell lines. The decreased cluster density of A673 cells in the presence of VN could indicate that it exerts an inhibitory influence on cell adaptation and growth [40]. However, the slight increase in cluster density observed in the HG with added VN cultured with the PDX73 cell line during the third week, contrary to what happens without added VN, suggests that this cell line may have specific adaptive or resistant mechanisms allowing a rebound in proliferation in the presence of VN [16]. These findings reflect a high variability in cell adaptation and behavior among cell lines. They can help to optimize culture conditions and better understand cellular interactions in these 3D environments.

Regarding the culture times studied, we observed that extending the time to 3 weeks produced smaller clusters of A673 cells in HGs, with large spaces between them and pre-apoptotic-like features, leading to loss of cell–cell and cell–HG adhesion. When evaluating density and area by cluster size, the A673 cell line showed differential behavior in the proliferation of large versus small clusters over time, as well as a marked reduction in cluster areas at 3 weeks independent of VN addition that could also be attributed to cell stress and death. Increased VN secretion has been associated with the bodily response to tissue damage or inflammation in studies with hepatic tissues, indicating that cells are trying to survive [62]. The higher VN release to culture media at 3 weeks compared to 2 weeks could be associated with this cell response to stress or to cell lysis. On the other hand, the relative stability in cluster density and area of the PDX73 cells along time, and its morphological observation in H&E indicate a better adaptation to the 3D models.

Cluster circularity analysis yielded interesting data regarding cellular migration. In the A673 cell line, clusters were round with few cell protrusions (circularity close to 1), especially in HGs with cross-linked VN, suggesting that this glycoprotein probably does not facilitate intercellular adhesion for easy migration of this cell line [63]. Evaluating PDX73 clusters, their low circularity, together with the VN detected in culture media over time, and the pericluster crown formation with interterritorial VN in HGs without added VN may be related to cell migration [62,64]. Moreover, when PDX73 was cultivated in HGs with added VN, low circularity and VN secretion was maintained, but we observed irregular pale halo formations around the clusters, lighter in anti-VN stain than the rest of the VN-HGs, which was particularly notable at 3 weeks. This finding could be linked to scaffold degradation by ES cells (i.e., by metalloproteinases, adamalysins, cathepsins, bone morphogenetic protein 1, and Tolloid-like proteinases, among others), suggesting that they are preparing genetic and epigenetic pathways to remodel their environment and promote cell migration [40,64,65]. This also supports the previously described role of VN in cell migration as it leads to cell attachment to and detachment from the ECM through its multiple binding sites. In other cancers, such as lung adenocarcinoma or ovarian carcinoma, VN has been shown to become a potent pro-migratory factor in plasma and other fluids, which, when released from its inhibitory complex with fibrinogen, facilitates cancer cell escape from the tumor, spread through the blood stream and lymphatic system, and subsequent metastasis to body cavities with low levels of fibrinogen [56,66]. Interesting future lines of study include further analysis of cluster and cell movement in our 3D models with and without added VN, also compared with cell VN secretion, to elucidate whether this pro-migratory capacity also affects ES cells.

## 5. Conclusions

In this study, we successfully cultivated two ES cell lines in simple, economical and quick-to-manufacture 3D models. The differing response between the A673 and PDX73 cell lines in 3D sf-GTA models with and without VN cross-linking highlights the importance of considering the specific characteristics of each cell line as a reflection of inter- and intratumor heterogeneity in patient samples. Both cell lines secreted VN, and cross-linking VN in HGs appeared to have a positive effect on cell growth in the PDX73 cell line. Studies with more cell lines (especially PDX cells and/or short-term primary cultures) and with co-cultures with stromal cells are ongoing to analyze whether added VN could influence tumor aggressiveness and thus be proposed as a biomarker. The 3D models presented in this study will allow significant progress in our understanding of ES at both molecular and clinical levels. Moreover, VN detection in liquid biopsies should be explored as a potential prognosis biomarker in ES patients.

Our findings could also have significant implications for designing therapeutic strategies (mechanotherapies) that modulate ECM–cell interactions, i.e., by blocking components such as VN in ES treatment. The well-known A673 cell line could be ideal for studies of basic molecular mechanisms and experiments that require stability and easy manipulation. In contrast, the PDX73 cell line would be more suitable for preclinical studies due to its closer fidelity to the patient’s original tumor, making it valuable for assessing treatment efficacy and developing personalized therapeutic strategies. A broader spectrum of cellular behaviors in different scaffolds composition along time would be useful to test and predict possible responses to therapies, especially mechanotherapies, in these 3D models.

We have provided a robust platform for investigating how ECM impacts tumor cell survival, progression, and migration. We have modulated its stiffness to study a specific element of the tumor ECM, VN, opening the possibility of its use to study other proteins of interest and other tumor types. As a new drug-testing platform, this approach paves the way for developing more effective and personalized therapeutic strategies. 

## Figures and Tables

**Table 1:** Cluster classification parameters.

Number of Cells	Classification	Color Code
<9	Small	Cyan
>10 and 38<	Medium	Green
>39 and 95<	Big	Yellow
>96 and 169<	Giant	Magenta
>170	Huge	Blue

## Data Availability

Readers who wish to obtain additional data or details related to this study are encouraged to contact the corresponding authors.

## Supplementary Materials

The following supporting information can be downloaded at: https://www.mdpi.com/article/10.3390/cancers16193347/s1, Section S1: Digital microscopic analyses of 3D models; Figure S1: Digital detection of hydrogel (HG) clusters and cells. (A) Example of an image of an HG after perimeter detection (green line) and cluster detection (green circles). (B) Cluster and cell detection: Example of an HG with clusters circulated in different colors according to the consensual cell number (Table 1) and cell detection in each cluster (red). (C) Out-of-cluster cell detection. The yellow borders represent the clusters. The red circles represent cells detected in the HG not belonging to any cluster; Figure S2: Vitronectin (VN) secretion of Ewing sarcoma (ES) and Neuroblastoma cell lines in 2D cell cultures. Detection of vitronectin secreted and number of cells in A673 and PDX73 from ES, and SH-SY5Y from NB as control. Green and orange bars represent the concentration of VN secreted to the culture media measured with the scale of ng/mL per million of cells (left Y-axis), and dots represent the number of cells quantified in the monolayer cultures measured with scale of millions of cells, as shown in the right Y-axis; Figure S3: Comparison of vitronectin (VN) levels secreted to culture media of 2D and 3D cultures by Ewing sarcoma cell lines (*p*-value = 0.044); Figure S4: Detection of vitronectin (VN) secreted to culture media by the cell lines A673 (A) and PDX73 (B) grown in hydrogels (HGs). Orange and blue bars represent concentration of VN secreted to the culture media measured with scale of the left Y-axis in ng/mL per million of cells and dots represent number of cells quantified in the 3D cultures measured in millions of cells (right Y-axis). Blue and orange bars represent HGs without (no VN) and with added VN (VN), respectively. Light colors refer to 2w culture and dark colors to 3w; Figure S5: Cluster density (clusters/nm^2^) in each hydrogel replicate divided by size (cell count inside), composition, and culture time. (A) A673 and (B) PDX73 cell line. Boxes in cyan represent no added VN in the scaffold (noVN), boxes in orange represent added VN in the scaffold (VN), a light color of both represents culture for 2 weeks (2w) and dark color of both represent culture for 3 weeks (3w). The number of clusters is at the top of each bar; Figure S6: Cluster density in 3D cultures of Ewing sarcoma cell lines (*p*-value < 0.0001). Table S1: Information obtained per hydrogel replicate; Table S2: Area of clusters vs. time and composition of hydrogels cultured with A673 cell line; Table S3: Area of clusters vs. time and composition of hydrogels cultured with PDX73 cell line; Table S4: Circularity of clusters vs. time and composition of hydrogels cultured with A673 cell line; Table S5: Circularity of clusters vs. size of clusters of A673 cell line; Table S6: Circularity of clusters vs. time and composition of hydrogels cultured with PDX73 cell line; Table S7: Circularity of clusters vs. size of clusters of PDX73 cell line.
