# Supplementary material for: Characterization of Vitronectin Effect in 3D Ewing Sarcoma Models: A Digital Microscopic Analysis of Two Cell Lines
Source: Cancers (Basel). 2024 Sep 30;16(19):3347. doi: 10.3390/cancers16193347 (PMC11476106; doi:10.3390/cancers16193347)
Supplement: Supplementary file 1 [file cancers-16-03347-s001.zip › cancers-3241417-supplementary.pdf]

Supplementary materials

Section S1: Digital microscopic analyses of 3D models

QuPhat[TH], and its extension StarDist, was used for all digital analyses. For digital analyses of 3D models, a semi-automatic method was developed to detect HG area, clusters and cells in the scanned H&E-stained slides.

The first step consisted of encircling the entire HG perimeter using a script based on the auto-threshold segmentation method. For HG detection with H&E staining, we used the Huang auto-threshold method by blue channel, with a minimum detection area of 90,000 μm^2 (Figure S1A). The previous tool was adapted to detect clusters using the Otsu auto-threshold method by hematoxylin channel with a minimum area of 500 μm^2 (Figure S1A), and a pretrained StarDist model for nucleus segmentation based on brightfield (i.e., hematoxylin, available in https://github.com), adjusted for details such as nucleus area and mean hematoxylin intensity, was used to distinguish and count the cells within each cluster (Figure S1B).

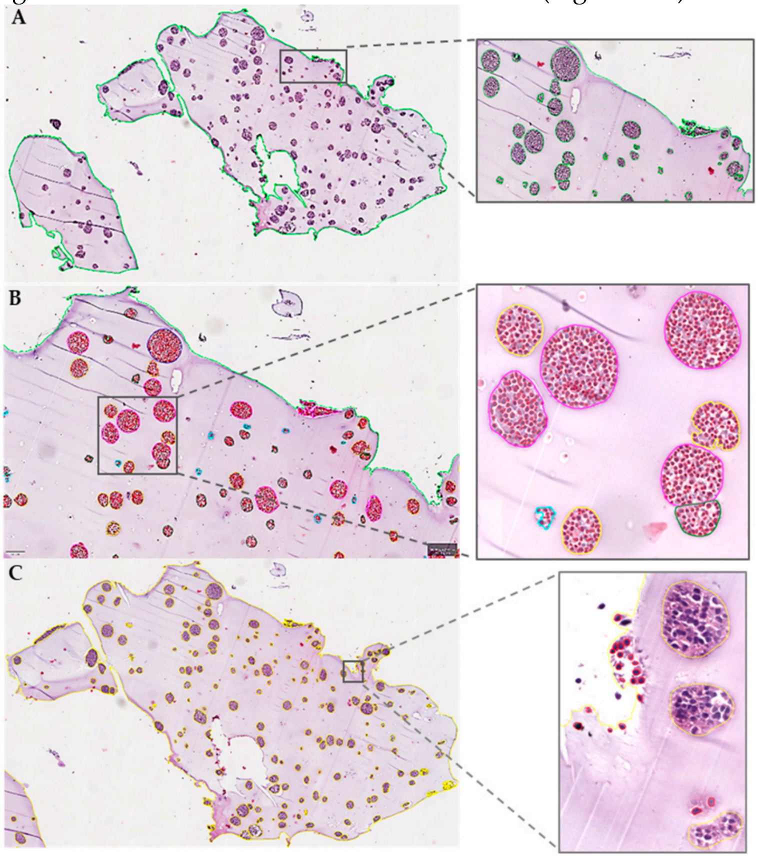

Figure S1. Digital detection of hydrogel (HG) clusters and cells. (**A**) Example of an image of an HG after perimeter detection (green line) and cluster detection (green circles). (**B**) Cluster and cell detection: Example of an HG with clusters circulated in different colors according to the consensual cell number (Table 1) and cell detection in each cluster (red). (**C**) Out-of-cluster cell detection. The yellow borders represent the clusters. The red circles represent cells detected in the HG not belonging to any cluster.

We used quartile analysis of the cell count in each cluster of the two cell lines studied and consensus was reached for size classification (Table 1). Next, a new script was adapted to classify clusters by cell number/size which was then validated by experts.

The last step was to detect single cells present in each HG not forming part of any cluster, and to combine them with the total number of detections inside clusters per HG obtained from the previous step (Figure S1C).

Figures and Tables

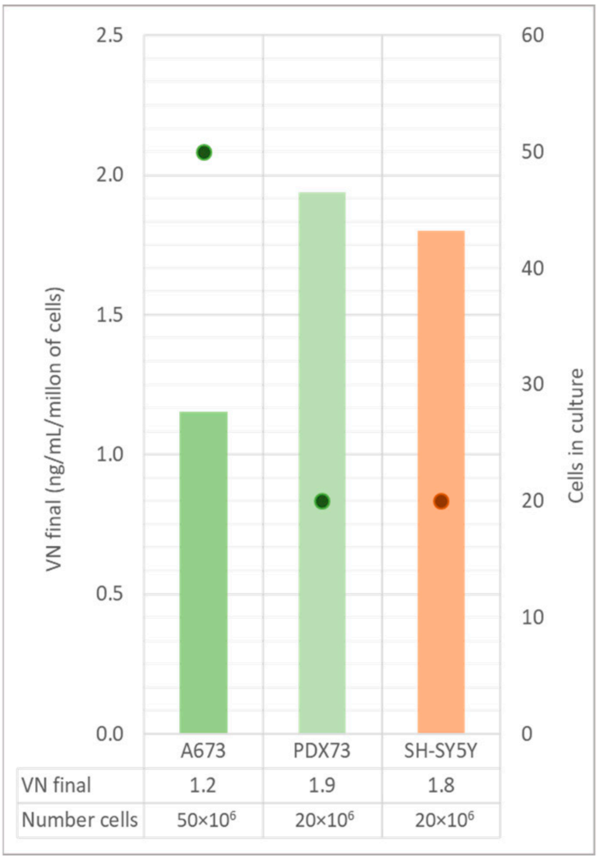

Figure S2. Vitronectin (VN) secretion of Ewing sarcoma (ES) and Neuroblastoma cell lines in 2D cell cultures. Detection of vitronectin secreted and number of cells in A673 and PDX73 from ES, and SH-SY5Y from NB as control. Green and orange bars represent the concentration of VN secreted to the culture media measured with the scale of ng/mL per million of cells (left Y-axis), and dots represent the number of cells quantified in the monolayer cultures measured with scale of millions of cells, as shown in the right Y-axis.

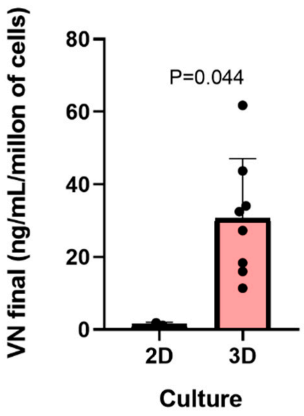

Figure S3. Comparison of vitronectin (VN) levels secreted to culture media of 2D and 3D cultures by Ewing sarcoma cell lines (p-value= 0.044).

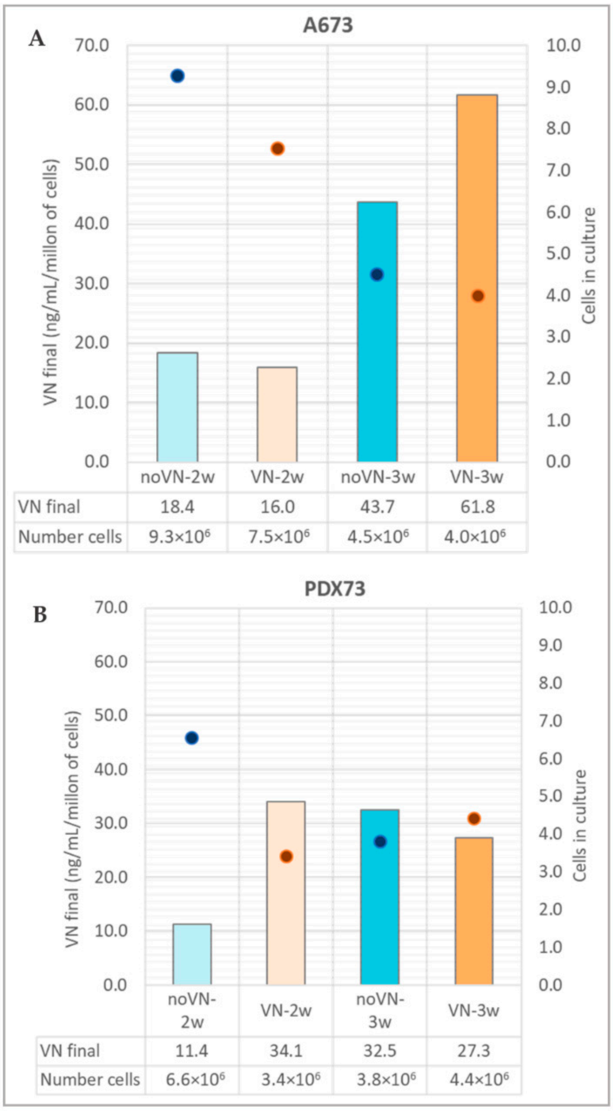

Figure S4. Detection of vitronectin (VN) secreted to culture media by the cell lines A673 (A) and PDX73 (B) grown in hydrogels (HGs). Orange and blue bars represent concentration of VN secreted to the culture media measured with scale of the left Y-axis in ng/mL per million of cells and dots represent number of cells quantified in the 3D cultures measured in millions of cells (right Y-axis). Blue and orange bars represent HGs without (no VN) and with added VN (VN), respectively. Light colors refer to 2w culture and dark colors to 3w.

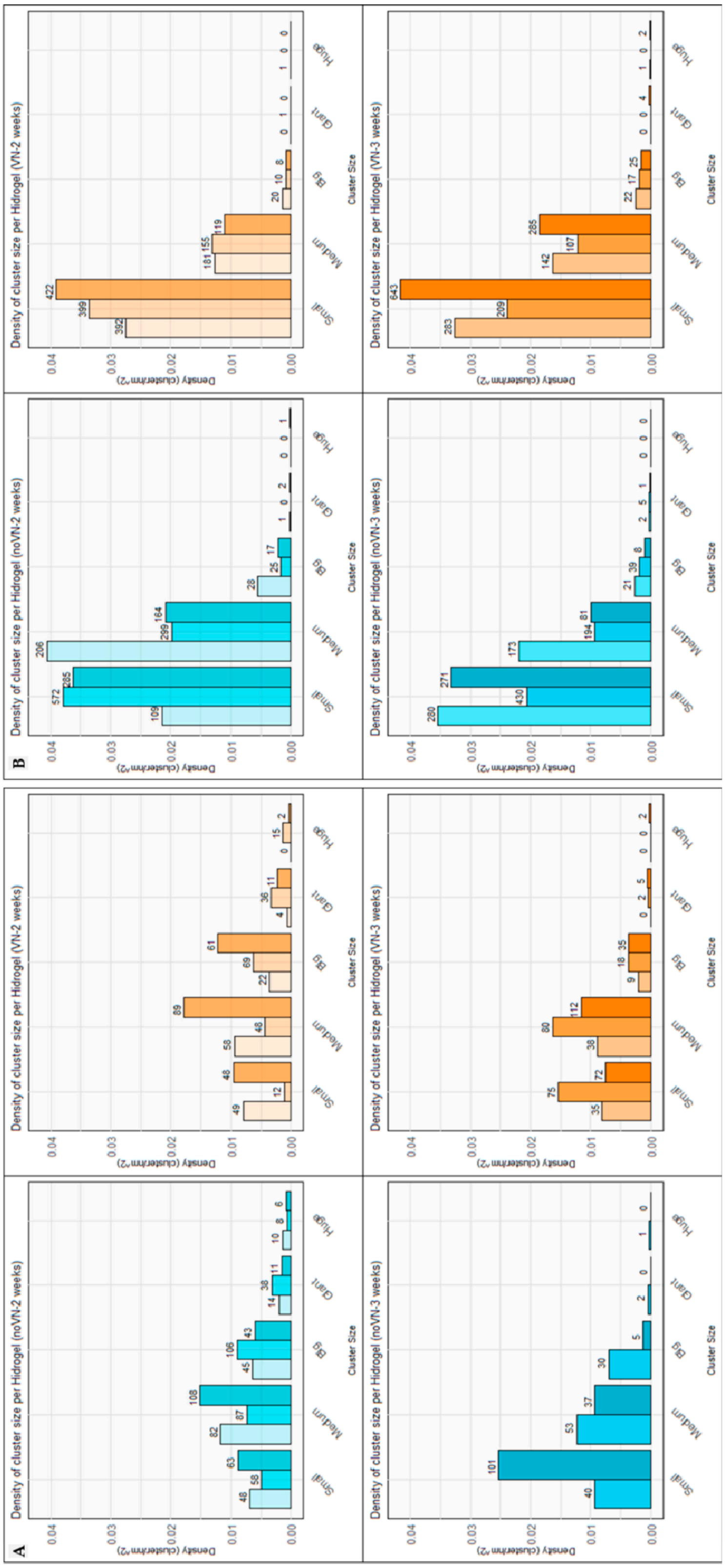

Figure S5. Cluster density (clusters/nm²) in each hydrogel replicate divided by size (cell count inside), composition and culture time. (**A**) A673 and (**B**) PDX73 cell line. Boxes in cyan represent no added VN in the scaffold (noVN), boxes in orange represent added VN in the scaffold (VN), a light color of both represents culture for 2 weeks (2w) and dark color of both represent culture for 3 weeks (3w). The number of clusters is at the top of each bar.

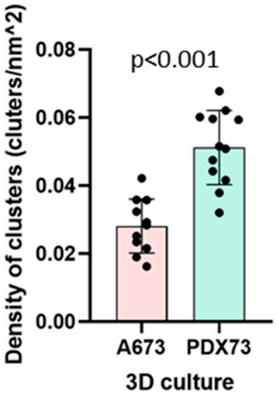

Figure S6. Cluster density in 3D cultures of Ewing sarcoma cell lines (p-value<0.0001).

Table S1. Information obtained per hydrogel replicate.

Line	Hydrogel	Scaffold composition	Time of culture	Hydrogel Size (cm^2)	Number of clusters	Number of cells	Cluster median area (μm^2)
A673	HG1	noVN	2 weeks	7.014	199	11573	1239.5
	HG2	noVN	2 weeks	11.81	297	15896	2606.7
	HG3	noVN	2 weeks	7.14	231	10552	1358.7
	HG1	VN	2 weeks	6.16	133	3664	936.31
	HG2	VN	2 weeks	11.08	180	15336	3464.65
	HG3	VN	2 weeks	5.003	211	8292	1385.7
	HG1	noVN	3 weeks	------	------	-------	------
	HG2	noVN	3 weeks	4.31	126	4115	1030.2
	HG3	noVN	3 weeks	3.99	143	1960	408.22
	HG1	VN	3 weeks	4.32	82	1945	925.085
	HG2	VN	3 weeks	4.89	175	3944	813.43
	HG3	VN	3 weeks	9.65	226	6470	1133.9
PDX73	HG1	noVN	2 weeks	5.08	344	7156	898.8
	HG2	noVN	2 weeks	15.09	896	11686	514.25
	HG3	noVN	2 weeks	7.86	469	7686	595.43
	HG1	VN	2 weeks	14.27	594	7688	579.75
	HG2	VN	2 weeks	11.89	565	6948	437.12
	HG3	VN	2 weeks	10.80	549	5689	339.06
	HG1	noVN	3 weeks	7.91	476	6450	457.49
	HG2	noVN	3 weeks	20.85	668	10013	547.55
	HG3	noVN	3 weeks	8.16	361	4434	432.88
	HG1	VN	3 weeks	8.69	448	6739	440.54
	HG2	VN	3 weeks	8.77	333	5103	509.06
	HG3	VN	3 weeks	15.45	959	11888	488.44

Table S2. Area of Clusters vs. time and composition of hydrogels cultured with **A673** cell line

		Interaction			
	**Statistical Data**	**VN – 2w** vs. **noVN - 2w**	**VN – 3w** vs. **noVN – 3w**	**noVN - 2w** vs. **noVN – 3w**	**VN – 2w** vs. **VN – 3w**
**Small**	N sample	109-169	182-141	169-141	109-182
	p-value	ns	<0.0001****	ns	0.0010***
**Medium**	N sample	195 -277	230-90	277-90	195-230
	p-value	ns	0.0451**	0.0017	ns
**Big**	N sample	152-194	62-35	194-35	152-62
	p-value	ns	ns	ns	0.0051
**Giant**	N sample	51-63	7-2	63-2	51-7
	p-value	ns	0.0215	ns	ns
**Huge**	N sample	17-24	2-1	24-1	17-2
	p-value	ns	ns	ns	ns

ns: not significant

Table S3. Area of Clusters vs. time and composition of hydrogels cultured with **PDX73** cell line

		Interaction			
	**Statistical Data**	**VN – 2w** vs. **noVN - 2w**	**VN – 3w** vs. **noVN – 3w**	**noVN - 2w** vs. **noVN – 3w**	**VN – 2w** vs. **VN – 3w**
**Small**	N sample	1213-966	1135-981	966-981	1213-1135
	p-value	ns	ns	0.0008	ns
**Medium**	N sample	455-669	534-448	669-448	455-534
	p-value	*	ns	ns	ns
**Big**	N sample	38-70	64-68	70-68	38-64
	p-value	ns	ns	ns	ns
**Giant**	N sample	1-3	4-8	3-8	1-4
	p-value	ns	ns	ns	ns
**Huge**	N sample	Insufficient	Insufficient	Insufficient	Insufficient
	p-value				

ns: not significant

Table S4. Circularity of Clusters vs. time and composition of hydrogels cultured with **A673** cell line

	Interaction				
	**Statistical Data**	**VN – 2w** vs. **noVN - 2w**	**VN – 3w** vs. **noVN – 3w**	**noVN - 2w** vs. **noVN – 3w**	**VN – 2w** vs. **VN – 3w**
**Small**	N sample	75-109	164-130	109-130	75-164
	p-value	<0.0001	<0.0001	ns	ns
**Medium**	N sample	137-172	200-77	172-77	137-200
	p-value	ns	<0.0001****	<0.0009***	ns
**Big**	N sample	133-164	54-28	164-28	133-54
	p-value	ns	ns	ns	ns
**Giant**	N sample	41-47	6-1	47-1	41-6
	p-value	ns	ns	ns	ns
**Huge**	N sample	Insufficient	Insufficient	Insufficient	Insufficient
	p-value				

ns: not significant

Table S5. Circularity of Clusters vs. size of clusters of **A673** cell line

	Interaction				
	**Statistical Data**	**noVN - 2w**	**VN – 2w**	**noVN – 3w**	**VN – 3w**
Small - Medium	N sample	109-172	75-137	130-77	164-200
	p-value	<0.0001****	ns	ns	ns
Small - Big	N sample	109-164	75-133	130-28	164-54
	p-value	<0.0001****	ns	0.0013**	ns
Small - Giant	N sample	109-47	75-41	130-1	164-6
	p-value	<0.0001****	ns	ns	ns
Small - Huge	N sample	109-16	75-17	Insufficient	Insufficient
	p-value	0.0005***	ns		
Medium - Big	N sample	172-164	137-133	77-28	200-54
	p-value	ns	ns	0.0038**	ns
Medium - Giant	N sample	172-47	137-41	77-1	200-6
	p-value	0.0271*	ns	ns	ns
Medium - Huge	N sample	172-16	137-17	Insufficient	Insufficient
	p-value	ns	ns		
Big – Giant	N sample	164-47	133-41	28-1	54-6
	p-value	ns	ns	ns	ns
Big – Huge	N sample	164-16	133-17	Insufficient	Insufficient
	p-value	ns	ns		
Giant - Huge	N sample	47-16	41-17	Insufficient	Insufficient
	p-value	ns	ns		

ns: not significant

Table S6. Circularity of Clusters vs. time and composition of hydrogels cultured with **PDX73** cell line

		Interaction			
	**Statistical Data**	**VN – 2w** vs. **noVN - 2w**	**VN – 3w** vs. **noVN – 3w**	**noVN - 2w** vs. **noVN – 3w**	**VN – 2w** vs. **VN – 3w**
**Small**	N sample	1157-927	1110-945	927-945	1157-1110
	p-value	<0.0001****	ns	ns	0.0002
**Medium**	N sample	434-634	513-420	634-420	434-513
	p-value	ns	0.0112	<0.0001****	<0.0001****
**Big**	N sample	36-62	54-47	62-47	36-54
	p-value	ns	ns	0.0079	0.0111
**Giant**	N sample	1-2	3-5	2-5	1-3
	p-value	ns	0.0154**	ns	ns
**Huge**	N sample	Insufficient	Insufficient	Insufficient	Insufficient
	p-value				

ns: not significant

Table S7. Circularity of clusters vs. size of clusters of **PDX73** cell line

		Interaction			
	**Statistical Data**	**noVN - 2w**	**VN – 2w**	**noVN – 3w**	**VN – 3w**
Small - Medium	N sample	927-634	1157-434	945-420	1110-513
	p-value	<0.0001****	ns	<0.0001****	ns
Small - Big	N sample	927-62	1157-36	945-47	1110-54
	p-value	ns	ns	0.0002***	<0.0001****
Small - Giant	N sample	927-2	1157-1	945-5	1110-3
	p-value	ns	ns	ns	0.0221*
Small - Huge	N sample	927-1	1157-1	Insufficient	1110-1
	p-value	ns	ns		ns
Medium - Big	N sample	634-62	434-36	420-47	513-54
	p-value	ns	ns	ns	0.0005***
Medium - Giant	N sample	634-2	434-1	420-5	513-3
	p-value	ns	ns	ns	0.0411*
Medium - Huge	N sample	634-1	434-1	Insufficient	513-1
	p-value	ns	ns		ns
Big – Giant	N sample	62-2	36-1	47-5	54-3
	p-value	ns	ns	ns	ns
Big – Huge	N sample	62-1	36-1	Insufficient	54-1
	p-value	ns	ns		ns
Giant - Huge	N sample	2-1	1-1	Insufficient	3-1
	p-value	ns	ns		ns

ns: not significant
